# Extraction camouflage treatment of a skeletal Class III malocclusion with severe anterior crowding by miniscrews and driftodontics in the mandibular dentition

**DOI:** 10.1186/s40001-025-02298-9

**Published:** 2025-01-23

**Authors:** Kai Zhang, Jiaojiao Li, Liyuan Yu, Wentian Sun, Kai Xia, Zhihe Zhao, Jun Liu

**Affiliations:** State Key Laboratory of Oral Diseases & National Center for Stomatology & National Clinical Research Center for Oral Diseases, Department of Orthodontics, West China Hospital of Stomatology, Sichuan University, Chengdu, Sichuan China

**Keywords:** Camouflage orthodontic treatment, Skeletal Class III malocclusion, Miniscrews, Driftodontics

## Abstract

An 18-year-old Chinese woman presented with chief complaints of crowded teeth and mild mandibular prognathism. Clinical and imaging examinations revealed a concave profile, a protruded chin, increased lower anterior facial height mild, skeletal Class III and Angle’s Class III malocclusion, with anterior crossbites, and crowded teeth. Extraction camouflaged therapy combined with miniscrews skeletal anchorage was employed to relieve crowding and retract the mandibular anterior teeth. The total active treatment time was 31 months. After treatment, functional occlusion and smile esthetics were significantly improved.

## Background

Skeletal Class III malocclusion is commonly characterized by a retrognathic and narrow maxilla, a prognathic and wider mandible, or a combination of both [1, 2]. Growth modification, orthodontic camouflage, or orthognathic surgery are main treatment choices to achieve a normal occlusion and improve facial aesthetics for skeletal Class III malocclusion. However, growth modification should be initiated before the pubertal growth spurt; beyond this stage, only orthodontic camouflage or orthognathic surgery are viable options [3].

Patients with skeletal Class III malocclusion generally exhibit dentoalveolar compensation, accompanied with proclination of the maxillary incisors and retroclination of the mandibular incisors [4], to adapt their craniofacial skeletal patterns and to achieve occlusal function [5]. For Class III malocclusion with severe skeletal discrepancies, the optimal treatment is generally orthodontics combined with orthognathic surgery. While for those who reject surgery and show a mild to moderate skeletal discrepancy with acceptable facial profile can benefit from compensatory orthodontic treatment by establishing an acceptable occlusion, allowing teeth displacement based on their supporting bone, and masking the underlying skeletal discrepancy [6, 7]. The treatment targets of presurgical orthodontic therapy and orthodontic camouflage treatment for nongrowing skeletal discrepancies are completely different, in which orthodontic decompensation and proper dentoalveolar compensation are crucial steps leading to successful treatment outcome, respectively [8, 9].

The goals of camouflage treatment involve achieving satisfactory alignment, function, and appearance by effectively utilizing dentoalveolar compensation to correct skeletal discrepancies [10]. The extent of dentoalveolar compensation is limited by the anatomic features of alveolar bone and remodeling potential of targeted teeth [11]. Overly compensatory proclination or retroclination of anterior teeth with insufficient alveolar bone would result in undesired fenestration or dehiscence. A reduced lower anterior facial height or a deep overbite can improve the prognosis in camouflage treatment for skeletal Class III malocclusion. This is because clockwise rotation of the mandible helps masking a prognathic mandible and enhances the concave profile, contributing to a more favorable overall outcome [12]. Therefore, when choose camouflage orthodontic therapy for skeletal Class III malocclusion, the following must be cautiously considered: (1) the severity of skeletal discrepancy and patients’ complaints; (2) vertical dimension; (3) the anatomic features of dentoalveolar bone.

The primary factors involved in the dental equilibrium are resting pressure from lip and tongue, extrinsic forces from habits or orthodontic appliances, dental occlusal forces, and eruption forces from periodontal membrane [13]. Due to tooth extraction, arch expansion, and the removal of oral bad habits during orthodontic treatment, the original equilibrium of the patient's stomatognathic system is broken, and the remaining dentition will naturally shift to establish a new dental equilibrium.

Physiologic drift was first proposed by Bourdet [14], which referred to the natural physical movement of other teeth in the dental arch after tooth extraction. In addition, the improved condition in tooth alignment resulted from spontaneous drift was called “physiological driftodontics” [15]. In some orthodontic techniques, physiologic drift was used to simplify the treatment. Alexander [16] straight wire technique suggested that after the extraction of four first premolars, maxillary teeth should be performed first, while mandibular teeth were supposed to bond brackets after natural adjustment for a period of time. The potential benefits of this period time of physiologic drift includes better occlusal relationship, increased dentoalveolar support, and a shorter overall time of orthodontic therapy owing to spontaneous realignment of the dentition [17].

This case report presents extraction camouflage orthodontic treatment using miniscrews and driftodontics to correct a skeletal Class III malocclusion with anterior crossbites and severe crowding. The treatment results were clinically acceptable, leading to enhanced smile aesthetics.

### Diagnosis and etiology

An 18-year-old Chinese woman presented to our hospital with primary concerns of dental crowding and mild mandibular prognathism. She reported no history of trauma or previous orthodontic treatments, no family history or bad oral habits.

**Fig. 1:**
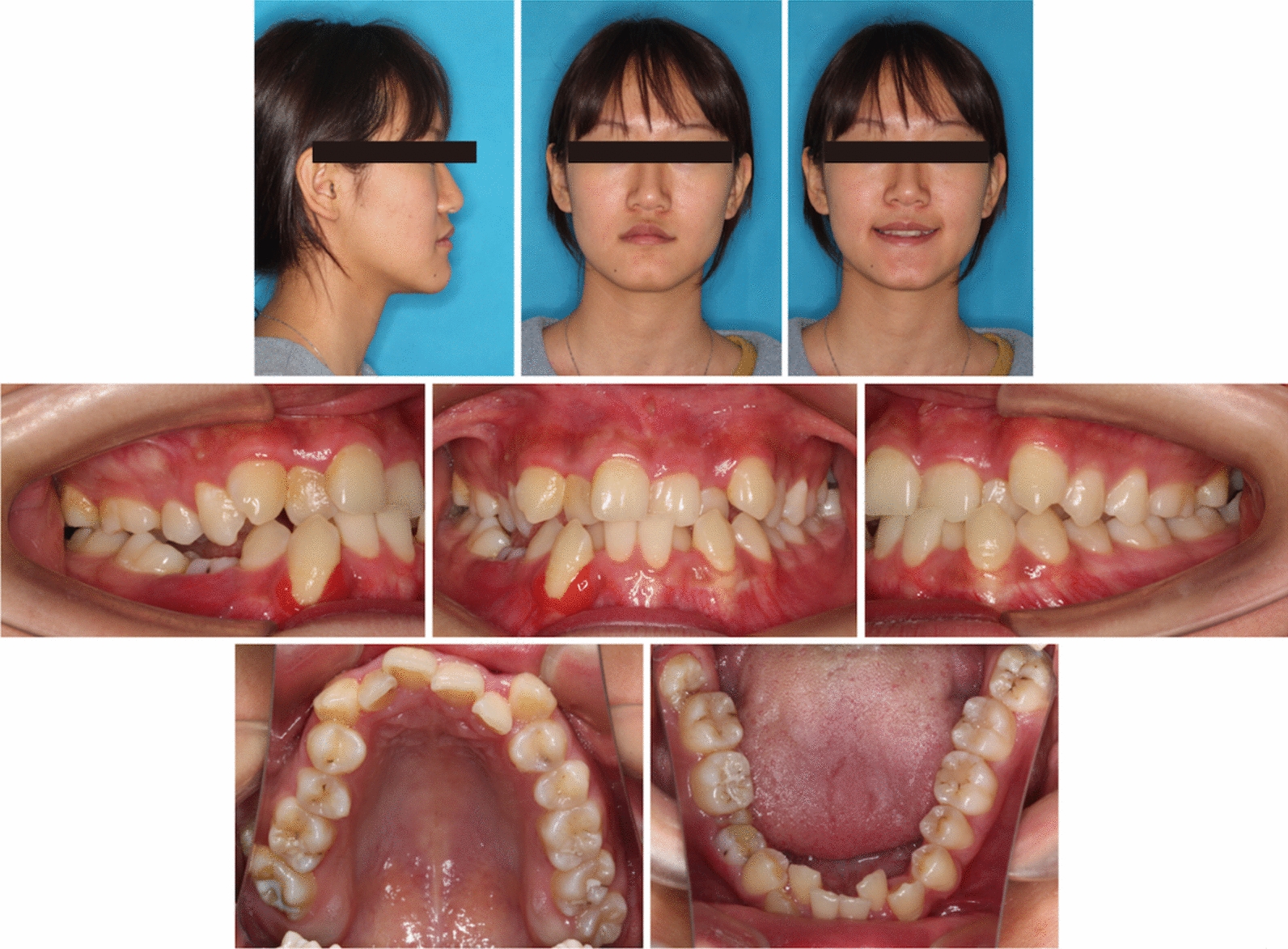
Pretreatment facial and intraoral photographs

**Fig. 2:**
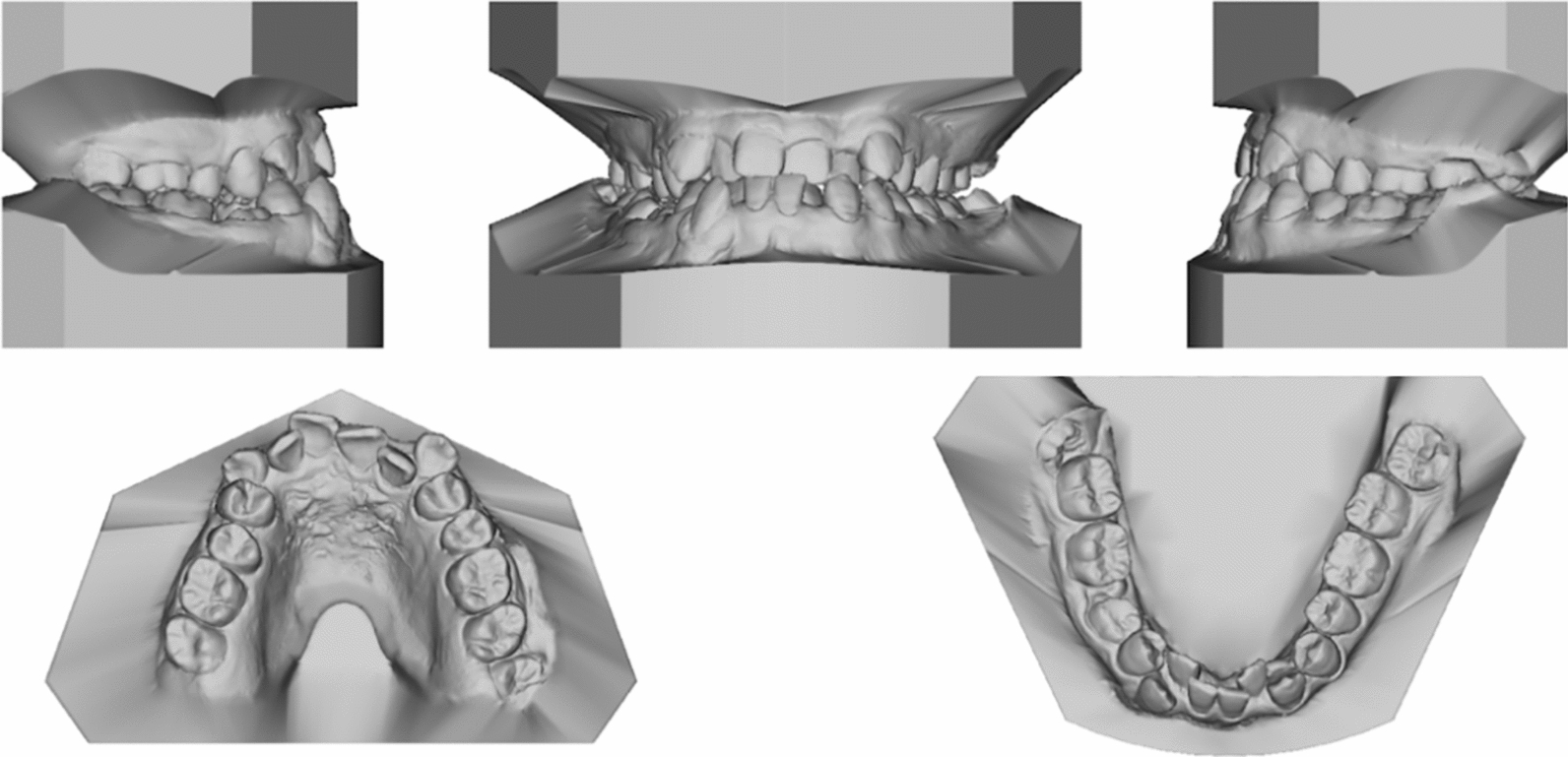
Pretreatment dental casts

Pretreatment facial photographs revealed a slightly concave profile, a protruded chin, and increased lower anterior facial height (Fig. 1). Nasolabial angle was acute (NLA, 79.3°), and both the upper and lower lips located behind the E-line (UL-EP, − 4.1 mm; LL-EP, − 2.2 mm). Mild facial asymmetry and decreased maxillary incisor exposure were observed. The maxillary dental midline was aligned with the facial midline, whereas the mandibular dental midline was shifted 1 mm to the right. The intraoral examination and study casts (Fig. 2) exhibited Class III molar and canine relationships with anterior crossbites and severe crowding. The mandibular right second premolar was impacted, and the deciduous mandibular right second molar was retained. Localized gingivitis observed, especially in anterior region. Cast analysis revealed a narrow maxillary dental arch, a mild curve of Spee (2.8 mm at right and 3.2 mm at left), severe crowding (9.7 mm of maxillary arch and 10.5 mm of mandibular arch), and normal value of Bolton index analysis (79.2% of anterior ratio and 91.1% of overall ratio).

**Fig. 3:**
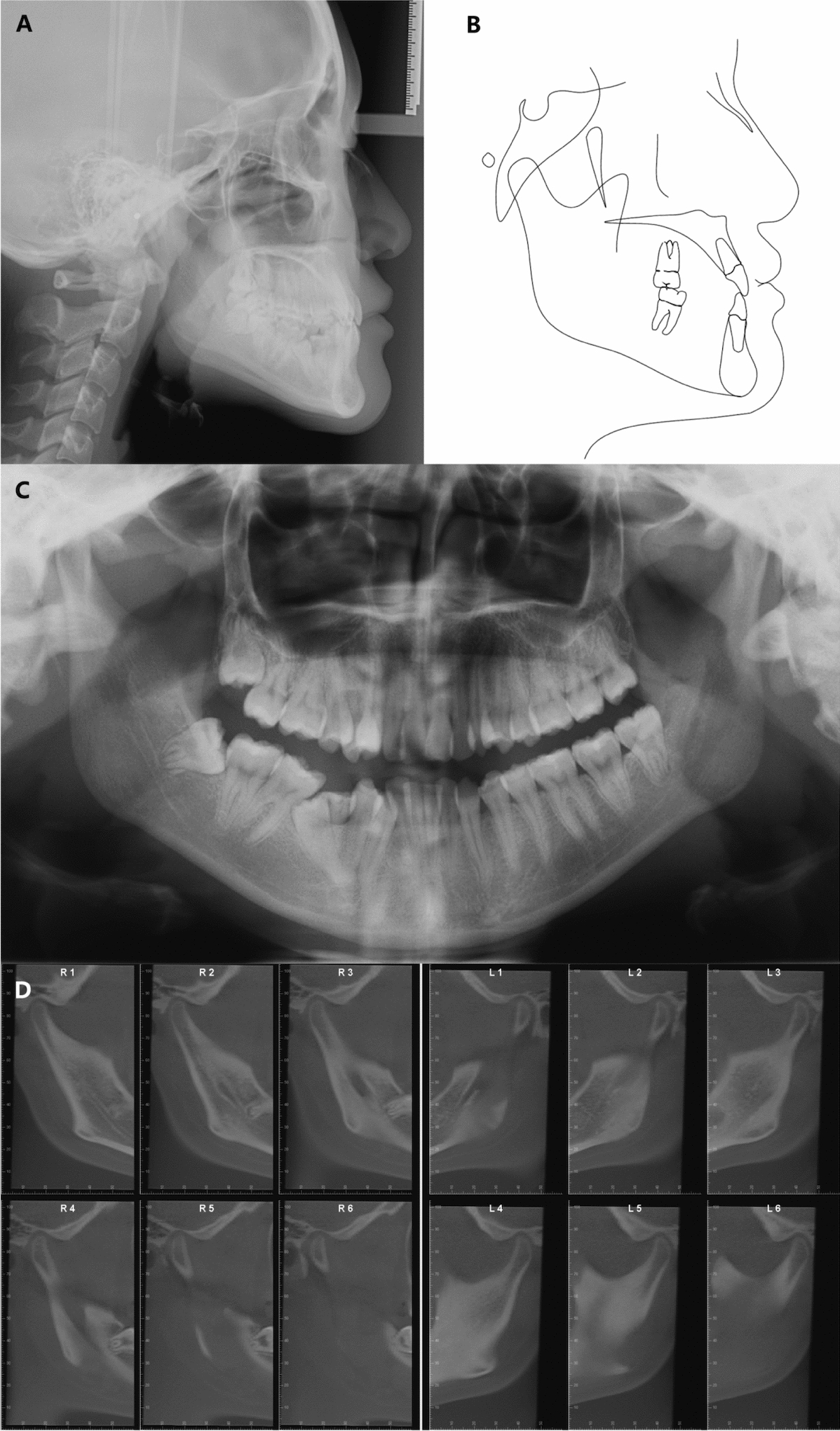
Pretreatment radiographs: **A** Lateral cephalogram. **B** Lateral cephalogram tracing. **C** Panoramic radiograph. **D** Cone-beam computed tomography image of both TMJs. L, left TMJ; R, right TMJ

Panoramic and cephalometric radiographs were obtained prior to the initiation of treatment (Fig. 3). Panoramic radiograph showed impacted mandibular right second premolar, retained deciduous teeth, and the presence of the maxillary and mandibular third molars. The lateral cephalometric analysis and tracing showed a mild skeletal Class III relationship characterized by a slightly prognathic mandible (ANB, − 0.4°; SNA, 84.9°; SNB, 85.3°) and average mandibular plane angle (SN–MP, 31.8°). The inclination of the maxillary incisors was within the normal range (U1-SN, 106.4°), while the mandibular anterior incisors exhibited compensatory retroclination (IMPA, 72.8°). The cephalometric measurements are listed in the Table 1. The evaluation of the temporomandibular joint (TMJ) (Fig. 3D) showed no signs of TMJ dysfunction, including pain, joint noise, limited jaw movement, or other related problems. Mesial proximal caries of maxillary right lateral incisor was observed in the cone beam computed tomography (CBCT) image.

**Table 1:** Pretreatment and posttreatment cephalometric analysis

Measurement	Norm	Pretreatment	Post-treatment
SNA°	81.7 ± 2.5	84.9	85.2
SNB°	78.9 ± 2.2	85.3	85.1
ANB°	2.8 ± 1.2	− 0.4	0.1
SN–MP°	32.9 ± 4.2	31.8	30.9
*Y*-axis°	63.5 ± 3.2	55.3	56.0
S–Go/N–Me	65.9 ± 3.8	68.4	68.6
ANS–Me/N–Me	53.3 ± 1.8	58.3	57.0
U1–L1°	123.2 ± 6.2	151.6	153.1
U1–SN°	105.1 ± 6.2	106.4	105.1
U1–NA°	23.3 ± 6.2	20.6	19.5
U1–NA (mm)	5.6 ± 3.6	3.7	1.8
L1–NB°	27.4 ± 4.7	15.3	6.9
L1–NB (mm)	5.8 ± 2.3	− 0.1	− 1.1
L1–MP°	95.4 ± 4.7	72.8	71.8
UL–EP (mm)	− 0.5 ± 1.9	− 4.1	− 3.5
LL–EP (mm)	1.3 ± 1.9	− 2.2	− 2.8

The patient was diagnosed with a mild skeletal Class III malocclusion, accompanied by dental Class III malocclusion, dental crowding, anterior crossbites, impaction of the mandibular right second premolar, and a retained deciduous mandibular right second molar.

### Treatment objectives

The treatment objectives were to (1) align and level the maxillary and mandibular teeth; (2) correct anterior crossbites; (3) coordinate the width of the maxillary and mandibular dental arch; (4) correct the midline of the mandibular dental arch; (5) establish Class I occlusal relationship with appropriate anterior overjet and overbite; and (6) eliminate crowding.

### Treatment alternatives

Two treatment options were provided to the patient. The first option was miniscrews-assisted camouflage orthodontic treatment with extraction of the maxillary first premolars, mandibular left first premolar, impacted mandibular right second premolar, retained deciduous mandibular right second molar and mandibular third molars. Miniscrews were designed as absolute anchorage to help retraction of mandibular teeth, and appropriate Class III traction was used to adjust molar relationship. The second option was orthodontic combined orthognathic treatment, with setback of mandible to correct the skeletal discrepancy and achieve maximum improvement of the facial esthetics. After careful consideration, the patient desired for non-invasive approach and chose camouflage orthodontic treatment.

### Treatment progress

Prior to bracket bonding, pulp treatment was first performed on the maxillary right lateral incisor. Then, maxillary first premolars, mandibular left first premolar, impacted mandibular right second premolar, retained deciduous mandibular right second molar and mandibular third molars were extracted. Self-ligation brackets (Damon-Q; Ormco Co, Brea, Calif) were bonded to the maxillary arch, and bite turbos were placed on the occlusal surfaces of the mandibular first molars to unlock the bite. The mandibular arch treatment was deferred until sufficient physiologic drift occurred in the mandibular teeth, alleviating the severe anterior crowding to some extent [16]. Both arches were aligned and leveled by sequenced 0.012, 0.014, 0.016, 0.016 × 0.022, 0.018 × 0.025-in nickel titanium archwires and 0.018 × 0.025-in stainless steel archwires.

In regards to mandibular anterior crowding, brackets were bonded on mandibular teeth except incisors after 6 months of physiologic drift. To provide maximum anchorage for retraction of mandibular anterior teeth, at 9th month of treatment, two miniscrews (12 mm, VectorTAS; Ormco Co, Brea, Calif) were inserted into the buccal alveolar bone bilaterally at the height of external obilque line positioned distally to the second molars in mandibular arch [18]. Two weeks after insertion, passive ligation was applied from mandibular right first premolar or left canine to the miniscrew respectively. Active space closure on both arches was commenced initially on 0.018 × 0.025-in stainless steel archwires. At 15th month of treatment, a retraction force was applied from mandibular canines to the miniscrews, for distal movement of mandibular canines with maximum anchorage. At 21st month of treatment, mandibular canines moved into proper position without remaining space in buccal segment. Mandibular incisors were bonded with brackets and integrated into active alignment. At 25th month of treatment, bite turbos were removed bilaterally after occlusal interference disappeared. In the 27th month of treatment, light Class III elastics were employed to achieve mesial movement of the maxillary molars, thereby coordinating the molar relationship.

**Fig. 4:**
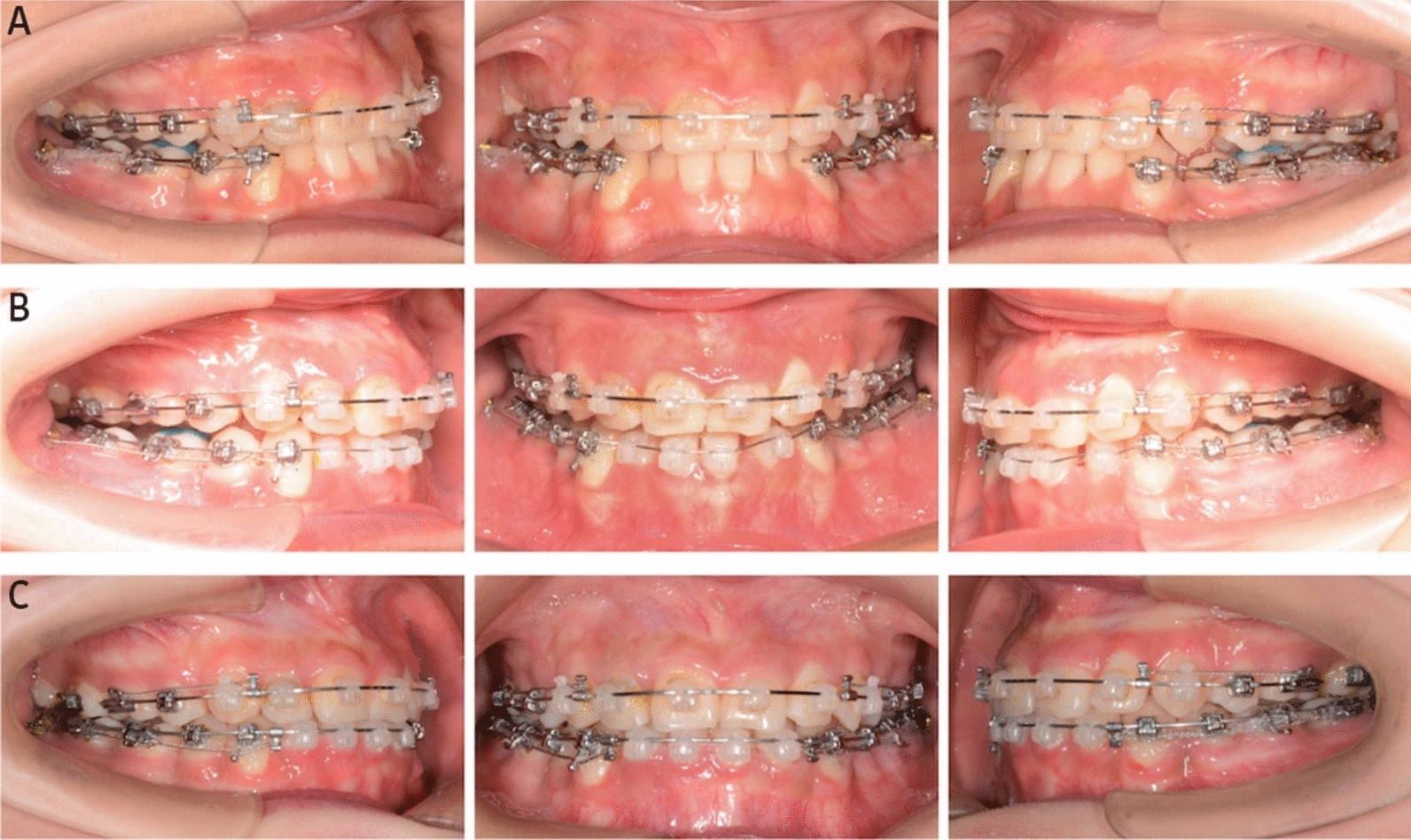
Treatment process: **A** at the 15th month of orthodontic treatment: retraction force was applied from mandibular canines to the temporary skeletal anchorage devices with segmented arch technic in mandibular dentition. **B** at the 21st month of orthodontic treatment: mandibular incisors were bonded with brackets and integrated into active alignment. **C** at the 27th month of orthodontic treatment: mandibular anterior teeth was retracted with sliding technique on 0.018 × 0.025-in stainless steel archwire

The overall duration of active treatment was 31 months (Fig. 4). The miniscrews were removed, and brackets were debonded. The extraction of bilateral maxillary third molars was recommended. Then, both clear retainers and Hawley retainers were provided, and the patient was instructed to wear clear retainers during the day and switch to Hawley retainers at night. In addition, she was advised to wear the retainers full-time for the first year, and then only at night thereafter.

### Treatment results

**Fig. 5:**
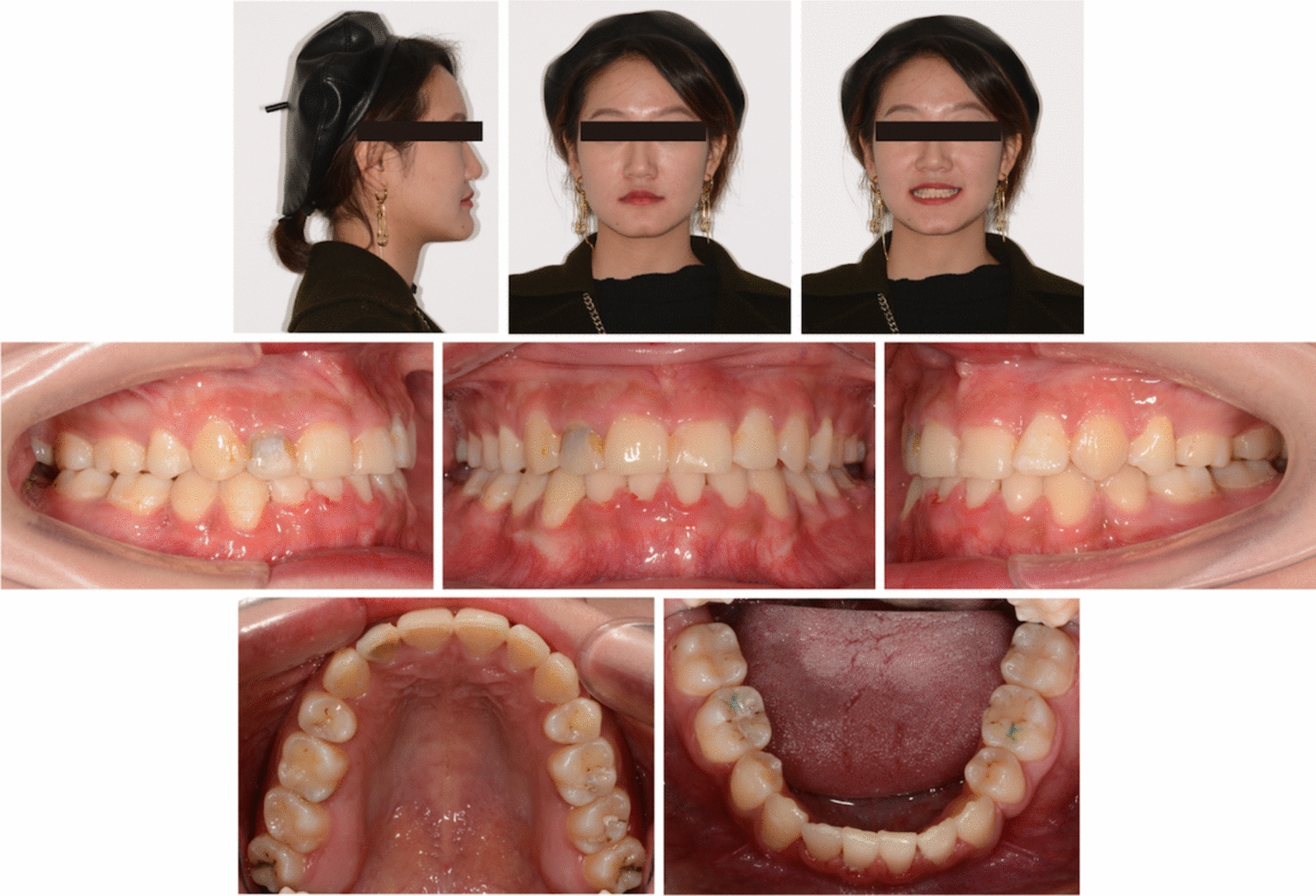
Post-treatment facial and intraoral photographs

**Fig. 6:**
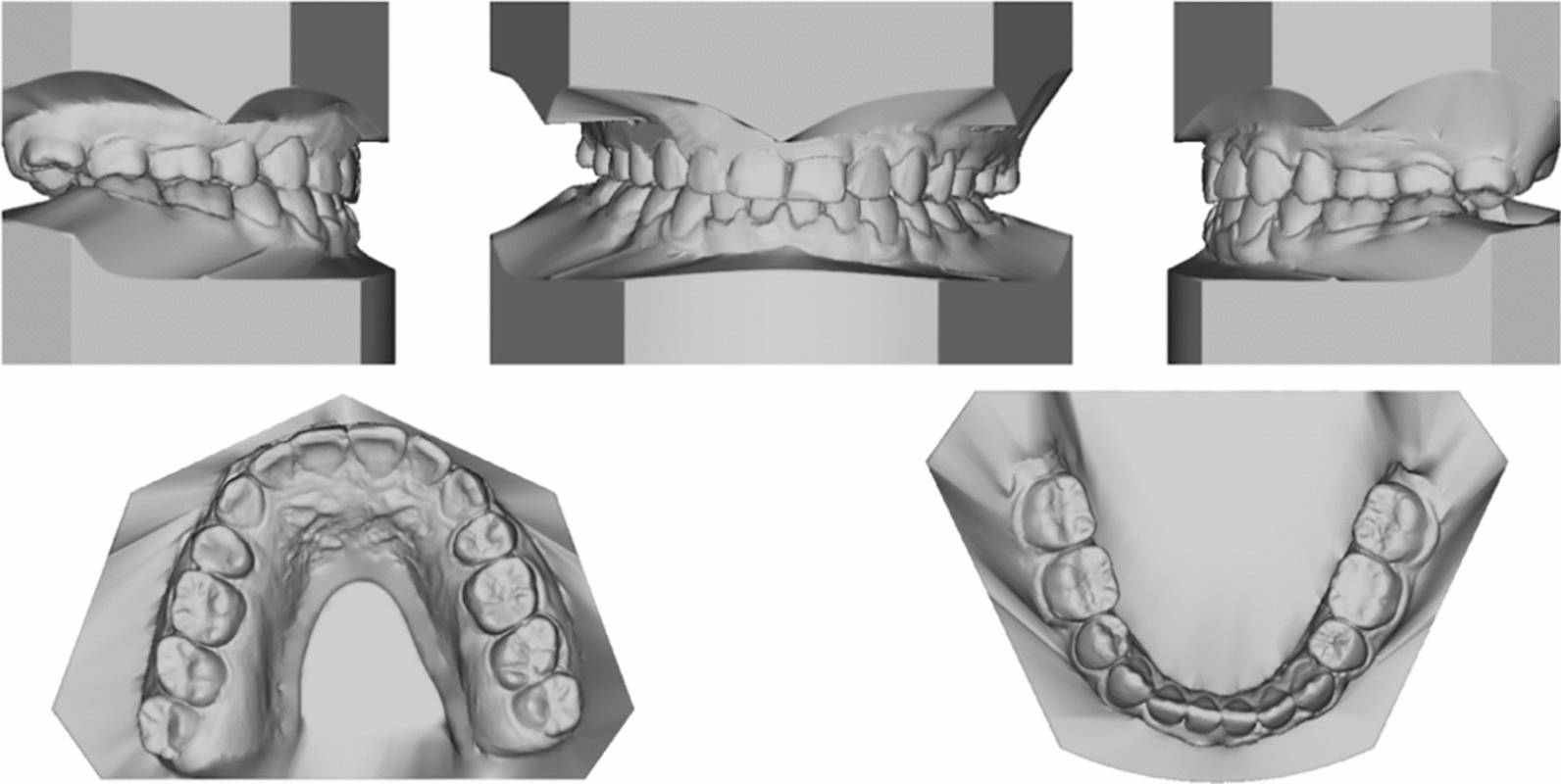
Post-treatment dental casts

The entire treatment spanned 31 months, and the patient was satisfied with the treatment outcome. Post-treatment intraoral examination (Fig. 5) and dental casts (Fig. 6) demonstrated that anterior crossbite was corrected, periodontal health was acquired, smile aesthetic was improved, the canine and molar relationships were aligned to achieve a Class I occlusion with proper overjet and overbite, the maxillary and mandibular dental midlines were aligned with the facial midline.

**Fig. 7:**
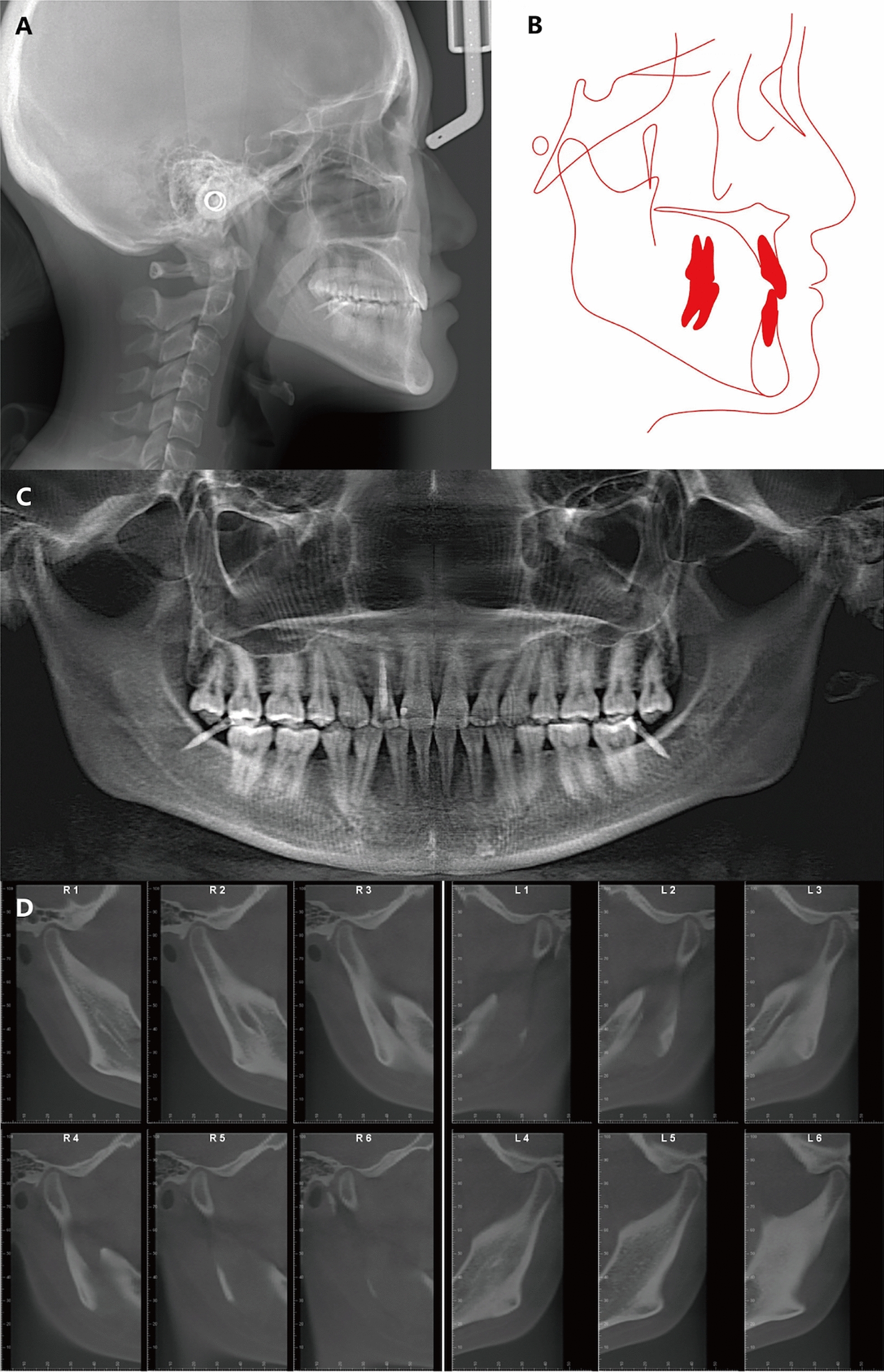
Posttreatment radiographs: **A** Lateral cephalogram. **B** Lateral cephalogram tracing. **C** Panoramic radiograph. **D** Cone-beam computed tomography image of both TMJs. L, left TMJ; R, right TMJ

**Fig. 8:**
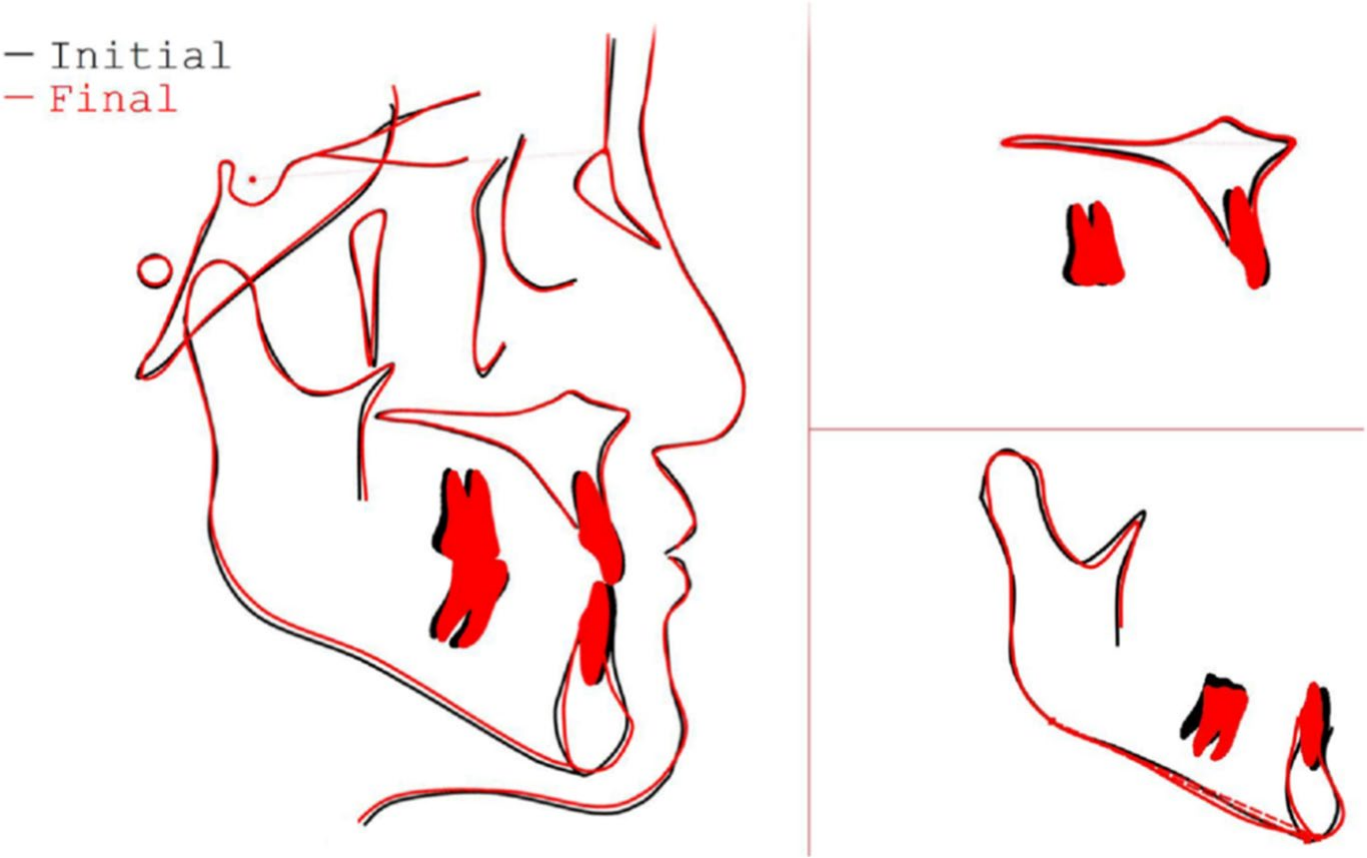
Lateral cephalogram superimposition tracings

**Fig. 9:**
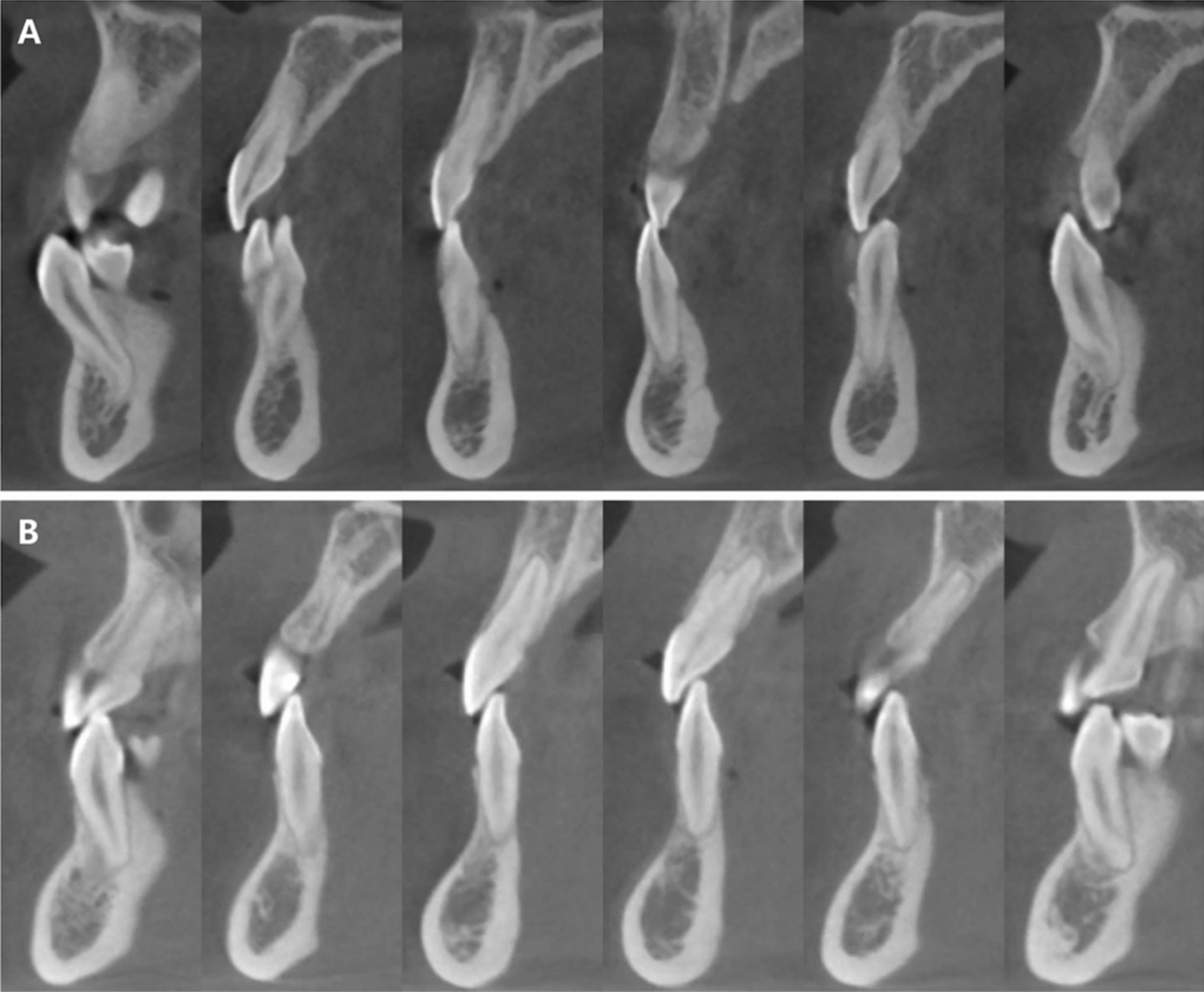
CBCT: Comparison incisors area at pretreatment (**A**) and posttreatment (**B**)

**Fig. 10:**
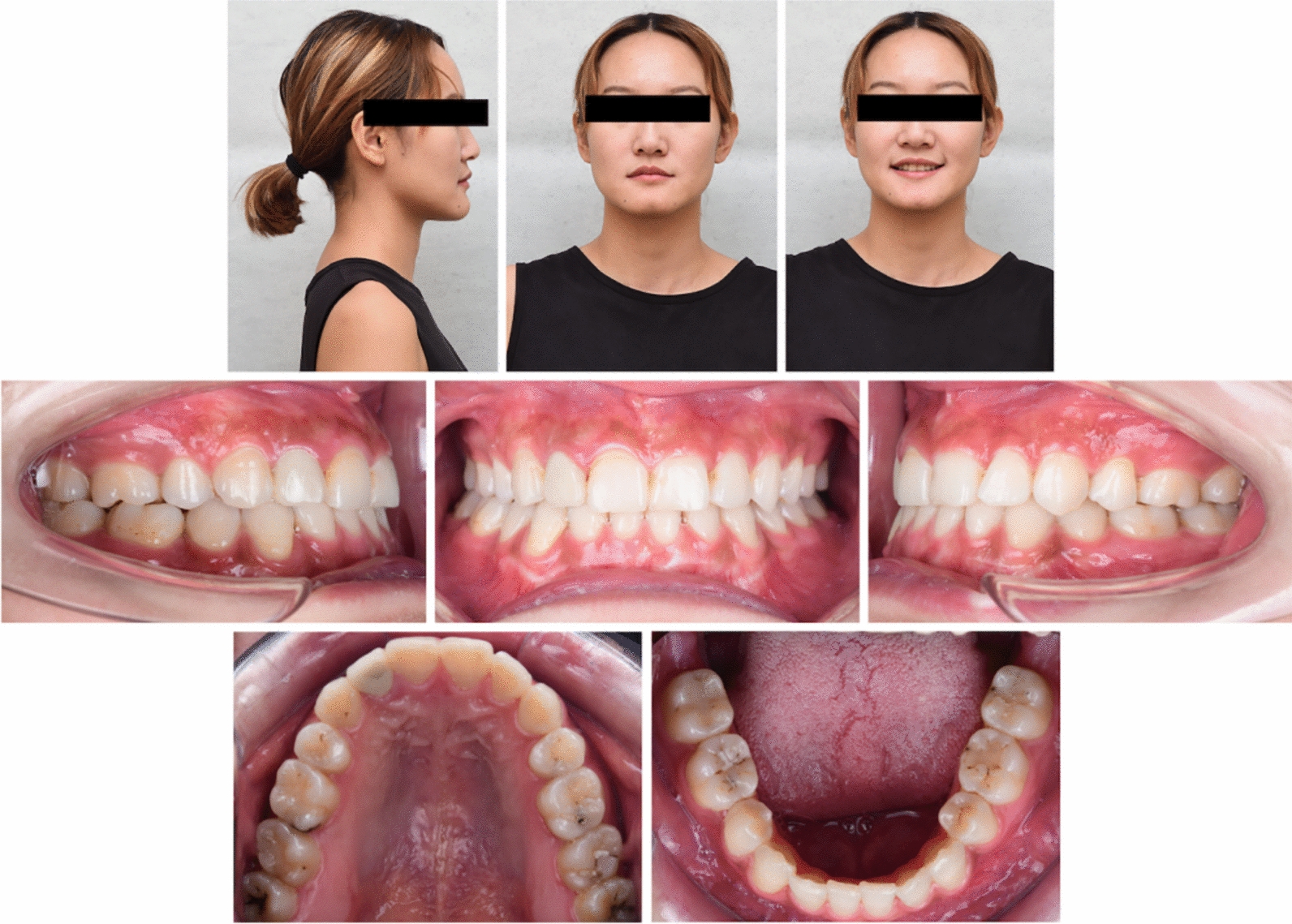
Follow-up facial and intraoral photographs after 2 years and 6 months of retention

The posttreatment panoramic radiograph revealed acceptable root parallelism in both arches, with no significant signs of root or bone resorption (Fig. 7C). Cephalometric analysis and superimposition (Figs. 7A, B and 8) indicated an improvement of sagittal skeletal discrepancy between maxilla and mandible (SNB, 85.1°; ANB increased from − 0.4° to 0.1°). In addition, the antero-posterior position of the maxillary incisors is basically unchanged, and the mandibular anterior teeth showed more lingual inclination (Fig. 9) (L1–NB, 6.9°; IMPA, 71.8°). The mandibular position was basically maintained as before treatment, and lower anterior height was not increased (SN–MP, from 31.8° to 30.9°). Furthermore, Lateral cephalogram superimposition showed no rotation of mandibular plane, which confirmed no rotation clockwise or counterclockwise of the mandible (Fig. 8). As for TMJ, CBCT revealed no harmful consequences of bilateral condyle, and no TMJ pain or discomfort was reported during or after the orthodontic treatment (Fig. 7D). Follow-up records of 2 years and 6 months are depicted in Fig. 10, showing that the occlusion remained stable.

## Discussion

When planning treatment for skeletal Class III malocclusion, the decision of whether to include orthognathic surgery is a key to a successful result. The severity of skeletal discrepancies in Class III malocclusion adult cases would decide whether the patient is suitable for orthognathic or camouflage treatment [19]. In general, orthognathic surgery is recommended to nongrowing patients with larger skeletal discrepancies, while camouflage therapy could be appropriate for milder discrepancies [3].

The pretreatment examination diagnosed the patient as a mild skeletal Class III malocclusion with a protruded chin, severe anterior crowding and an increased lower facial anterior height. Acceptable periodontal condition, mild skeletal discrepancies and less demanding of dentoalveolar compensation demonstrated that the patient would have a good prognosis by receiving camouflage orthodontic treatment. However, it is important to pay attention to vertical control in the process of orthodontic treatment [20]. If the jaw rotates clockwise post-treatment, it would be helpful to improve mandibular prognathism and mask the skeletal sagittal discrepancy. However, this could increase the lower facial height, resulting in a long-face appearance. If the jaw rotated counterclockwise, it would worsen mandibular prognathism, resulting in more unaesthetic profile. In this patient, the extraction space was primarily utilized to relieve crowding and retract the mandibular anterior teeth. Two miniscrews positioned distally to the mandibular second molars were applied as absolute anchorage to retract mandibular teeth, avoiding worsening Class III occlusal relationship because of posterior teeth mesial movement. As Class III traction for coordination of molar relationship was applied only for 2 months using light force, the extrusion amount of maxillary molar was negligible. The post-treatment outcome showed that the mandible and the occlusal plane were in the same position as before treatment without rotation, and the lower anterior facial height was not increased. Therefore, the camouflage planning solved the patient’s complaints and improve smile aesthetic without worsening unaesthetic profile.

In the “element II” of “Six Elements of Orofacial Harmony”, Andrews proposed the use of forehead position and angulation as a reference for the ideal position of the maxilla and defined the esthetic target as the facial surface of the maxillary central incisor tangent to the goal anterior limit line (GALL) [21, 22]. The anteroposterior position of the GALL shifts based on the angle of the forehead. In the Chinese Han population, the GALL moves forward to align with the glabella due to a steep forehead angle. The pretreatment analysis indicated that the facial point of the maxillary central incisors was primarily positioned at the GALL, suggesting it would maintain the position of the maxillary central incisors. In this case, maxillary extraction space was designed to relieve crowding and to promote mesial movement of maxillary molars, and post-treatment outcome showed good control of maintaining antero-posterior position of maxillary central incisors as pretreatment.

An important concept of orthodontic treatment is to improve oral health. The health and stability of periodontal tissue is the basis for achieving safer and more efficient orthodontic effect, serving as a vital foundation for achieving aesthetic, functional, and stable results in treatment [23]. In this patient, the mandibular anterior teeth were misaligned with gingival recession, gingivitis, especially mandibular right canine. Vasconcelos et al. [24] suggested that The compensatory retroclination of mandibular incisors in Angle Class III cases did not show a higher incidence of gingival retraction; however, the presence of existing gingival recession heightened the risk of further severe gingival recession. Maintaining optimal gingival health in the mandibular anterior region throughout orthodontic treatment is crucial for preventing the development of gingival recession. To avoid further gingival recession and improve periodontal condition, mandibular teeth were not bonding immediately after extraction and they were allowed to drift distally under physiological conditions. Early active alignment of the anterior crowding teeth in Class III relationship patients would result in round tripping movement, aggravating bone resorption and being detrimental to compensatory treatment. Driftodontics allows anterior teeth to move spontaneously towards extraction area, relieving the extent of severe crowding and beneficial to align the dentine. The rate of spontaneous space closure following the extraction of mandibular premolars was more rapid during the initial 6 month period, and later space closure had continued at a remarkable consistent rate [25]. In this case, the mandibular teeth were allowed to drift during the first 6 months of orthodontic treatment. In addition, when mandibular incisors was prepared for brackets bonding, the mandibular extraction space had been reduced significantly and the periodontal condition of mandibular anterior teeth had been improved. The post-treatment mandibular first molars showed no mesial movement compared to the pretreatment position. The use of miniscrews as maximum anchorage played a significant role in this outcome. Tooth self-adjustment promotes more physiologic tooth movement and is easier to maintain. It may lower the likelihood of relapse, reduce time spent in treatment, and improve periodontal health. Therefore, when cases are carefully selected, physiologic drift can be an effective method for clinical use.

## Conclusions

This case report illustrates the successful camouflage treatment of a skeletal Class III adult patient with anterior crossbites and severe crowding using miniscrews and driftodontics. With reduced cost, low technical sensitivity, and wide applicability, this treatment protocol provides a viable alternative for adult patients with skeletal Class III malocclusion.

## Data Availability

No datasets were generated or analysed during the current study.

## Abbreviations

CBCT: The cone-beam computed tomography
GALL: The goal anterior limit line
TMJ: The temporomandibular joint
